# Altitude and the distributional typology of language structure: Ejectives and beyond

**DOI:** 10.1371/journal.pone.0245522

**Published:** 2021-02-05

**Authors:** Matthias Urban, Steven Moran

**Affiliations:** 1 Center for Advanced Studies ‘Words, Bones, Genes, Tools’, University of Tübingen, Tübingen, Germany; 2 Institute of Biology, University of Neuchâtel, Neuchâtel, Switzerland; Leiden University, NETHERLANDS

## Abstract

The first decades of the 21st century have witnessed a renewed interest in the relationship between language structure and the various social and ecological niches in which the languages of the world are used and against the background of which they evolved. In this context, Everett (2013) argued for direct geographical influences on the sound structure of languages. It was observed that ejective consonants, produced with a sudden burst of non-pulmonic air to a salient acoustic effect, tend to occur in high-altitude environments in which these sounds may be adaptive due to a reduced articulatory effort and/or to prevent desiccation. Here, we evaluate this claim and at the same time place it into a broader context. We observe that the distribution of another class of typologically unusual sounds, uvulars, is highly similar to that of ejectives, but that the proposed explanations are not available to account for the similar geographical patterning of uvulars. Hence, we test an alternative explanatory account that would posit indirect rather than direct environmental influences on language structure that are mediated by anthropological factors, in particular the relative sociolinguistic isolation of speech communities at the highest altitudes. Applying Bayesian Logistic Mixed Effects Regression to a large database of phonological inventories of the world’s languages, however, we do not find strong support for either a correlation of ejectives or uvulars with high-altitude environments, though the association is somewhat stronger for ejectives than uvulars. A phylogenetic exploration of the development of both classes of sounds in two large language families spoken in widely different environments, Indo-European and Sino-Tibetan, together with a qualitative assessment of the dedicated literature, in contrast, suggests a strong role of language contact rather than environmental factors.

## Introduction

In the context of the rise of cultural evolution as a framework for studying cultural change, the present century has witnessed a surge of a strand of research which claims that human languages, like the human genome (e.g. [1, 2]), are adaptive to their respective environments broadly construed (see [3–6] for review). The relevant environmental niches to which languages are theorized to be adaptive to are of different kinds: they may be of a sociolinguistic kind—as when languages with many L2 learners respond to this role by evolving structures that increases their learnability by adults (see e.g. [7–9]). They may be related to the medium in which language is transmitted. For instance, syntactically more complex phrases and sentences tend to occur in the written rather than the oral medium, and more generally in languages with long traditions of writing and literacy [10]. Finally, a particularly vibrant yet not entirely uncontroversial strand of research within this broader context pertains to the relationship between languages and the physical environment in which they are spoken (compare, for example [11–14]). That such research should be a topic that is in the news again would have seemed unlikely just a couple of decades ago, as it was more or less banned from serious inquiry during the 20th century [15]. This is in large part in response to a preceding phase in which it blossomed in the 18th and 19th century. For example, in the context of so-called “climatic theory”, scholars of the times theorized a more or less direct relationship between languages and their respective environments and argued for various theories that range from the absurd (such as the idea that the perceived “harsh” sound of Swiss German results from the goiters of the Swiss that in turn are caused by the hard Swiss water [16]) to the moderately more plausible but speculative (e.g. the idea that “[T]he serrated close way of Speaking of Northern Nations, may be owing to their Reluctance to open their Mouth wide in cold Air, which must make their Language abound in Consonants; whereas from a contrary Cause, the Inhabitants of warmer Climates opening their Mouths, must form a softer Language, abounding in Vowels” [17, pg. 153-154]). In the 21st century, researchers have begun to rediscover the relationship between language structure and physical environment as a serious topic of inquiry, and have posited dependencies between certain features of languages, in particular in phonology, that are sometimes surprisingly similar to what was claimed in the 18th and 19th century (see [13] for extensive review of both phases in which such research flourished).

A facilitating role in the renaissance of the topic is played by the increasing availability of large linguistic databases that furnish comparative data across hundreds or even thousands of languages in readily accessible formats—though with the methodological danger of spurious correlations that arise only because of the vast possibilities of statistical hypothesis testing which these databases allow for [18]; see now [19] for an approach that mitigates that danger. Current approaches to linguistic typology are likewise conducive in paving the way towards the revival of this line of inquiry: typology has become less interested in the traditional goal of establishing linguistic types and (implicational) universals of language structure, but rather seeks to understand the distributions of typological features across time and space and their geographical, social, and historical determinants. In the context of this reorientation, it is becoming increasingly clear that typological features, especially but not exclusively rare ones, are distributed unevenly across the globe and the task of linguistic typology in its current orientation is to explain why these are distributed the way they are (see [20] for the programmatic statement).

The present contribution fits squarely into this context. Concretely, we seek to shed additional light on the possible role of the geophysical environment in which languages are spoken on the sound structure of human languages, which has been a central arena of theorizing in both phases in which research on the language-environment interface flourished. Specifically, we investigate one major claim regarding a possible effect of the environment on phonological structures that has been put forward recently, and put it into a broader empirical and theoretical context. The case concerns so-called ejective consonants, which have been theorized to have a non-random distribution in the languages of the world that is governed by the altitude at which they are spoken [21]. On the one hand, on the empirical side, we explore to what extent ejective consonants are the only class of sounds whose distribution can be theorized as depending on altitude. Concretely, we investigate whether uvular consonants are distributed similarly in the spoken languages of the world, as impressionistic evidence suggests; if so, this would constitute evidence that there are broader patterns with scope not only over the distributions of ejectives, but also other classes of rare and articulatorily costly sounds in the languages of the world. Everett [21] has suggested some possible ways in which ejectives may be adaptive in high-altitude environments that is based on the specific articulatory mechanisms that are involved in producing them. Since uvulars are articulatorily different from ejectives, available explanations for a possible altitude-dependent distribution that have been forwarded for ejectives do not apply. Therefore, on the theoretical side, we explore whether an alternative explanation that is capable of accounting for the distribution of both classes of sounds feasible. Rather than assuming a direct impact of the environment on speaker behavior, this account would posit an influence of the environment on social and economic behavior of people in high-altitude environments that then, indirectly, shapes linguistic distributions.

In the following section, we review in more detail the literature with which our contribution engages and from which the goals of our analyses emerge. Then, we discuss the properties of the data on which we base our analyses, and present their results. On the basis of a large database of phonological inventories, we find that the evidence for altitude as a predicting factor is generally weak for both classes of sounds, but slightly better for ejectives than for uvulars. However, a combination of a phylogenetic analysis of the development of each sound class, and a concomitant in-depth survey of the dedicated literature in historical linguistics, suggests that for both classes of sounds, contrary to both competing hypotheses, language contact is usually a major factor in generating their observed distributions. We discuss this finding and to what extent it can be reconciled with either of explanatory account in our final reflections in the concluding section.

## Altitude and the distributional typology of language structure: Ejectives and beyond

One prominent claim in the double context of the renewed interest in extralinguistic determinants on languages as adaptive systems—especially environmental influences on linguistic structure beyond the lexicon—and the reorientation of linguistic typology has been made by Everett [21]. This study has raised considerable interest, but also controversy, especially because it makes the strong and explicit claim that there is a *direct* influence of the environment on language structure, which is, as the author argues, not culturally mediated as is assumed in alternative frameworks [11, 22].

**Fig 1:**
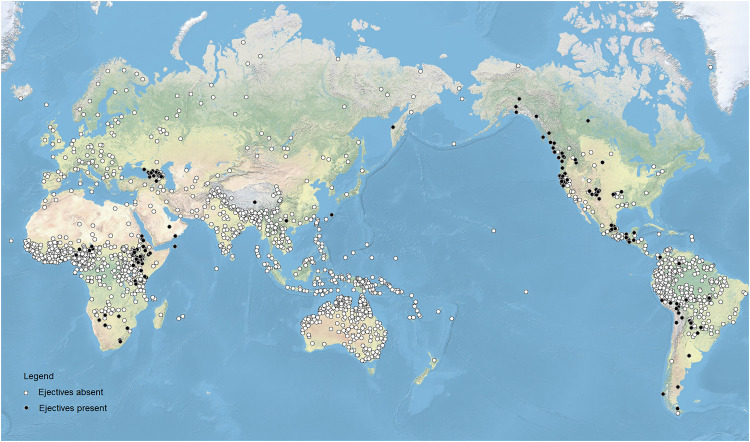
Languages with ejective consonants in the PHOIBLE database.

The case pertains to ejective consonants. Relatively rare in the phonological systems of the languages of the world, ejective consonants are unlike the majority of consonants because they are produced using a non-pulmonic airflow mechanism. Due to the simultaneous closure of the glottis and a secondary closure in the buccal cavity (at either one of the usual places of articulation, though for reasons of articulatory effort most frequently at the velum) an amount of air is “trapped” in the part of the vocal tract between the two closures. Raising of the glottis due to muscular contraction causes the local air pressure in the relevant part of the vocal tract to rise. This is often visible from the exterior by a characteristic movement of the Adam’s Apple. Upon sudden release of the closures, the air is released and the air pressure differential is rapidly equalized with an often salient acoustic effect that can be described impressionistically as a “pop”-like sound. To explore the distribution of ejective consonants, Everett [21] used data provided by the World Atlas of Language Structures (WALS [23], now updated as [24]), reducing the information provided in WALS to a binary variable (presence vs. absence of ejectives). The dataset includes information on 567 languages and is based on the earlier UCLA Phonological Segment Inventory Database (UPSID; [25, 26]). Everett observed that ejectives tend to cluster in or near high-altitude regions of the world, including the Andes, the Caucasus, and the African Rift valley. Fig 1 shows the occurrence of ejective consonants in the most recent version of the PHOIBLE database [27], which includes the UPSID data, but goes significantly beyond this seminal sample. It currently has data on phonological inventories from 2,186 distinct languages.

Impressionistically, the distribution of ejectives according to PHOIBLE is quite similar to that in WALS, with particularly salient hotspots in western North America, Mesoamerica, the Andes in South America, the Ethiopian highlands in North Africa, and the Caucasus in Eurasia.

To assess whether the distribution of ejectives responds to altitude as the visual impression suggests, Everett [21] obtained geographic point coordinates (latitude and longitude) as provided by the WALS database. Points are mostly chosen such that they correspond to the center of a language’s range, though for some languages idiosyncratic choices have been made (e.g., choosing a geo-coordinate near the historical center of distribution). In turn, Everett used these coordinates to obtain a measure for the altitude associated with each language in the sample.

Descriptively, the result was that the mean altitude (as extracted on the basis of WALS coordinates) for languages with ejectives was 955 meters above sea level (MASL) and that without them it was at 631. To investigate further, Everett defined “high altitude zones”, i.e. regions “greater than 1500m in altitude, plus land within 200 km of such a region of high altitude” (while defining “high altitude zones” as those at 1,500 MASL or greater, reference to Cohen and Small [28] is made, but no rationale for the decision to include a 200km perimeter is initially given). The resulting 2 x 2 classification (presence vs. absence of ejective consonants and location of a language within such a “high altitude zone” vs. location outside) showed a statistically significant difference; later in the analysis, Everett [21] demonstrates that languages with ejectives that are outside high altitude zones, thus defined, are closer on average to such regions than those without.

For quite some time, it has been recognized that both genealogical and areal biases must be taken into account when assessing typological distributions. Everett [21] does address the possibility that the distribution of ejectives and the impression that they tend to occur at higher altitudes may be influenced by particular language families. However, he outrightly dismisses the idea that genealogical biases might confound the results by observing that, in those regions where ejectives cluster together, it is always several language families that are represented and that contribute to the apparent pattern. Concomitant with the pioneering observation of large-scale areality in word order regularities, it has been suggested that a typological correlation can be considered genuine if it occurs in five of six so-called “macro areas”, which have since then become a sort of widely applied quasi-standard for assessing areal effects in evaluations of typological distributions and correlations [29]. Not situating his own analysis in this context explicitly, Everett offers a similar analysis by calculating the differences separately for Africa, Eurasia, South America, and North America, noting that the expected differential occurs in Africa, Eurasia, and South America but not in North America (however, the difference is significant only for Africa in [21], but it does come out as significant throughout on the basis of a larger analysis informally offered in [30]; in both cases, no correction for multiple testing appears to have been carried out).

Everett [21] offers two different theories as to how the inclusion of ejective sounds in phonological inventories may be adaptive in high-altitude environments, without making a commitment whether either, both, or neither is really operative [30]. One possible factor that Everett discusses is related to a tradition in linguistics that emphasizes the competing motivations, especially in phonology, between minimizing articulatory effort and maximizing expressive possibilities (e.g. [31]). Given that ambient air pressure is undoubtedly lower in high-altitude environments than it is at or near sea level, the articulatory effort of ejectives, specifically the creation of the pressure differential that is necessary for their production, should be lower at high altitudes. This may be an incentive for speakers of languages in high-altitude environments to use this acoustically salient class of sounds. On the other hand, Everett hypothesizes, ejectives may also be adaptive in high-altitude environments because the non-pulmonic airflow that is involved in the production of ejectives may help to prevent dehydration through the loss of water vapor with exhaled air, a significant problem in high-altitude environments due to generally lower air humidity (and the often limited availability of fresh water sources).

Evaluations of Everett’s [21] analysis and argument were quick to follow the original publication, though in the form of blogposts rather than contributions to peer-reviewed outlets. These responses have focused on replication of the association rather than on addressing the plausibility of the proposed mechanisms, and conclusions were divergent. While Roberts [18] reports approaching the proposal with considerable skepticism, to his surprise he in fact found support for the statistical association of ejectives with altitudes, whereas Hammarström [32], on the basis of a different statistical approach, could not replicate a significant association. Given the conflicting conclusions, and the informal manner in which they have been published, the question is neither conclusively confirmed nor conclusively rejected. Since evaluation has been mainly focused on methodological issues, and the significance of the association itself, it is natural that the examination of the proposed explanatory theories and the evaluation of these against possible alternatives have been put aside.

**Fig 2:**
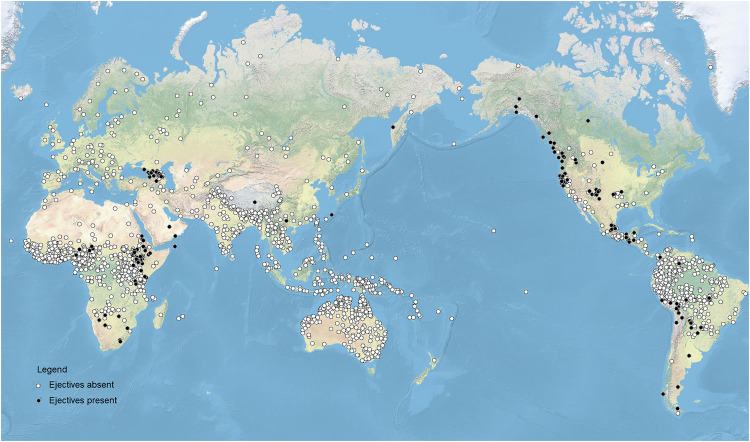
Languages with uvular consonants in the PHOIBLE database.

While working on a typology of American languages [33]), the first author of the present study noted impressionistically that ejectives in the languages of Middle and South America tend to co-occur with another type of relatively rare and articulatorily costly class of sounds, the so-called uvulars. Checking the American situation against large-scale phonological databases beyond the narrow situation in Middle and South America, it becomes clear that, in fact, the distribution of uvulars seems highly similar to that of ejectives. Fig 2 plots the location of languages with uvular consonants in the PHOIBLE database, giving a visual impression of this apparent distributional overlap.

In fact, on the basis of an initial visual impression one might be similarly inclined to suspect an altitude-dependent distribution of uvulars. The fit seems to be even *better* than that of ejectives, because “[t]he only major region of high altitude where languages with ejectives are absent is the large Tibetan plateau, along with adjacent regions of high altitude” [21], but uvulars actually are common even there (see discussion in Section **Analyses**).

The impressionistically similar distribution of uvulars in geographical space, with hotspots of occurrence in high-altitude mountain areas of different areas of the world, if genuine and robust, would also invite the search for hypotheses that could account for the distribution of both classes of sounds and their apparent overlap. Since the articulation of uvulars, like most other consonants (but unlike ejectives), involves pulmonic airflow, the explanations put forth by Everett [21] for the special case of ejectives are unavailable to explain a possible altitude-dependent distribution of uvulars in the languages of the world. However, if it can be corroborated, similarities in the distribution of both types of sounds would suggest that some general factor is in play and governing their distributions. This factor could be related to physical geography, or the environment broadly, but it does not necessarily have to be so. For instance, general mechanisms of expanding and saturating phoneme inventories, e.g., system-internal factors that are not yet understood, could very well be involved.

Here, we explore a particular alternative hypothesis that does make reference to physical geography, but in a more indirect fashion that is mediated by sociolinguistic patterns of language use. Mountain environments with their challenging topography are commonly thought of as having the effect of isolating human communities and limiting communication between them. This, in turn, is thought to influence the structural profiles of languages spoken in high-altitude environments, which, in the absence of significant second language learning that may exert simplification pressures, are thought of as accumulating complex and more generally rare and hard to learn structures. This can happen either through the retention of such structures that are lost elsewhere or by their accumulation through diachronic change (see [14] for review on language use and structure in mountain environments). Independently of Everett [21], Nichols [34, pg. 38] makes reference to precisely the classes of sounds of interest here when exemplifying this line of thought:

isolation favors (or at least does not disfavor) complexity, mountain geography favors isolation, and complexity of sound systems necessarily entails expansion along certain dimensions. Thus ejectives and uvulars can be found in mountain areas—not because harsh mountain geography deterministically causes language to add harsh consonant series (!), but because isolation favors complexity

However, what can legitimately count as “complex” segments? If “complexity” is for practical purposes equated with cross-linguistic rarity [35], then both classes of sounds qualify (though the question of how to precisely define “cross-linguistically rare” remains, cf. [36]). But also other possible manners of defining the controversial notion of complexity in language would lead to similar conclusions. For example, while complexity need not necessarily map one-to-one to articulatory effort, it is undoubtedly high for ejectives. However, a greater articulatory effort of uvulars vis-à-vis other pulmonic stops, which has been suspected before [37, pg. 268], is now biomechanically also quantifiable precisely. In terms of articulatory effort, uvulars (voiced and in consonant-vowel (CV) syllables) are only exceeded by retroflex consonants [38, pg. 79]; and in fact, voicing is disproportionally more costly for back consonants, [39, pg. 666-667]. Another possible piece of evidence for the marked status of uvulars is that, in situations of language death, where marked segments tend to merge with less marked ones in favor of the latter’s phonetic properties, uvular stops tend to merge with velars [40, pg. 60-61].

An additional reason for thinking in this direction comes from a study on affricate-rich languages in Eurasia [41]. As consonants with secondary articulation that require precise control over supralaryngeal aperture, affricates traditionally are considered as complex segments [42], and in affricate-rich languages, also cross-linguistically rare types of affricates occur. The distribution of affricate-rich languages is strikingly similar to that of languages with ejectives and uvulars. All three tend to be found in mountainous areas, affricate-rich languages especially in the Caucasus, the Hindu Kush, and the eastern Himalayas. Particularly rich inventories of affricates are typically dependent upon the presence of typologically uncommon retroflex affricates [41, pg. 574-576]. And since affricate-rich languages have much the same distribution as ejectives and uvulars, the distribution of affricate-rich languages in Eurasia would likewise be consistent with an account in terms of sociolinguistic behavior that is shaped by topography and that, in turn, shapes languages distributions, but inconsistent with direct environmental influences on language structure (Everett [30] addresses a similar scenario which has been suggested to him in personal communication).

Everett is, of course, right that implosives—on the basis of a visual inspection of maps (e.g. [43])—appear to exhibit a quite different geographic patterning [44]. However, since there is a preliminary reason to think that certain types of rare and articulatorily costly sounds, other than ejectives (e.g., uvulars), actually do correlate with high altitude—but others are, as Everett points out, less likely to (e.g., implosives)—we thus consider the preliminary and subjective assessment as a sufficient incentive to begin exploring more formally for which sound classes and types this holds true.

Under this hypothesis, then, the distribution of ejectives would turn out to be a special instance of a more general phenomenon that governs the distribution of (certain types of) complex segments in the languages of the world. Concomitantly, a theory that invokes the sociolinguistic dynamics of mountain areas would have greater explanatory power than Everett’s account by invoking a single principle that is capable of explaining the distribution of cross-linguistically rare types of sounds that involve either pulmonic or non-pulmonic airflow. Such an account would be in-line with the Boasian tradition, which assumes that the physical environment in which a language is spoken may shape the communicative needs and behavior of its speakers. And, if anything, these communicative needs and speech behavior, in turn, rather than the environment directly, would shape the structure of languages (see [15, 45] for original statements and [12] for a recent reappraisal in the context of the resurgence of research at the language-environment interface sketched in Section **Introduction**). Thus, in contrast with Everett’s [21] proposal, which explicitly argues for direct influence of the environment on language structure, if existent at all, it would be at best indirect, and mediated through human behavior.

From this discussion, a two-fold goal emerges for the present study. First, by formally reassessing the proposal of an altitude-based distribution of ejective consonants in the languages of the world, we seek to shed further light on these initial findings. At the same time, we offer several improvements in terms of the data used for the analysis and the procedures to arrive at sound results. Some of these improvements are due to reactions regarding the original article paper (e.g., [32], where the validity of using the WALS data as the basis of statistical analysis is questioned), and others are brought up in the context of discussions of other research on the relationship between language and environment (e.g., the necessity to control for genealogical and areal dependencies in the data in the same analysis rather than separately [46], or to bear in mind the potentially significant role of language contact in shaping segment distributions [47]). By reassessing the validity of the association between ejectives and altitude through a different dataset and by using different analytical techniques, we also contribute to a recent plea for robustness and incremental approaches in the assessment of specific hypotheses relating to the adaptive potential of human language [19].

However, we also emphasize that our study is not meant as a mere critical re-evaluation of Everett [21] in a narrow sense. By examining the distribution of uvular consonants in light of the same predictive parameter, i.e. altitude, and in the same overarching analytical framework, the main goal of our study is in fact to identify the underlying causes for the distribution of certain sound classes. Generally, research in this area has focused on sophisticated assessments of the distributions of individual phonological features. While these studies give us a good indication of the degree to which the distribution of the linguistic features can be thought of as governed by properties of their surroundings, where there is still a long way to go is in coming up with linguistically and culturally plausible concrete pathways by which the environmental influences might actually find their way into language structure. At the same time, it is notable that analyses have, for the most part, focused on individual classes of segments or phonological properties such as sonority [48] and tone [49, 50]. As the preceding discussion of Everett’s [21] claim for an altitude-dependent distribution of ejectives shows, this runs the danger of not seeing broader patterns that might affect the distribution of several heterogeneous sound classes or phonological features—as is the case for ejectives and uvulars, for instance. However, the joint consideration of these might induce, or indeed require, different and broader explanatory accounts that have scope over the distribution of both sound classes. Thus, at the same time as reassessing the relationship between ejectives and consonants, we seek to discriminate between the original direct environmental explanation for the distribution of ejectives and an alternative one that is more general in nature and based on indirect sociolinguistic effects of geography on linguistic structure rather than direct environmental ones.

## Data and coding

### Phonological data

One aspect of Everett’s [21] study that is criticized is the use of the UPSID-based WALS data [23], which was not designed for statistical evaluation, and, in line with current best practice, it has been recommended instead by Hammarström [32] to use either the World Phonotactics Database [51] or PHOIBLE [27]. Indeed, in a rejoinder to criticisms, Everett [30] provides summary statistics on the altitude of isolates and other analysis on the basis of data from the World Phonotactics Database. Since this source has in the meantime become unavailable, for our analyses in the present paper we use phonological data from the PHOIBLE database. The current version of PHOIBLE [27] contains phonological inventories from 2,186 distinct languages. Some of these inventories were extracted from primary descriptions of the languages for the purposes of inclusion in PHOIBLE, but it also incorporates data from other, typically more regionally specialized, phonological inventory databases. Using PHOIBLE’s system of classifying segments on the basis of distinctive features, we extracted the number of ejectives and uvular segments in the inventories from the database. For uvulars, we have taken into account consonants of all manners of articulation, but in a supplementary analysis we have also restricted uvulars to obstruents and excluded rhotics. For ejectives, we have taken into account all places of articulation. We have excluded segments of both types that were annotated as being marginal in the language in the primary analysis. For our main analysis, we later converted these numbers into a binary variable that merely registers the presence or absence of ejectives and uvulars consonants per language, following in this regard the original study on ejectives by Everett [21]. However, we also retained the original numeric counts in order to assess the relationship between altitude not only for the sheer presence or absence of both classes of sounds, but also the number of ejectives and uvulars in languages spoken at different altitudes.

Given that PHOIBLE incorporates data from heterogeneous sources, the 2,186 PHOIBLE languages map onto a significantly larger number of 3,020 inventories from different sources. These often provide divergent accounts on the phonological inventories of the described languages. Therefore, a measure to avoid the inclusion of a single language more than once in the analysis, which would occur by a direct analysis of the PHOIBLE data, is necessary. Where for a given language (as represented by the ISO 639-3 code) more than one inventory from different sources is available, we have selected one on the basis of the following hierarchy of sources (for a description of each source, see: https://phoible.org/contributors):

PH > GM > SAPHON > UZ > EA > ER > SPA > AA > RA > UPSID

This particular hierarchy was chosen because it maximizes the one-inventory per doculect principle, i.e. tertiary databases like SPA and UPSID often contain multiple references for “single” languages, which were typologized by the source creators. This hierarchy also maximizes the inclusion of contrastive tone because the UPSID database lacks descriptions of tone in its segment inventories.

After the selection of datasets according to this procedure, sometimes what remained in the highest-ranked source was data from more than one dialect of a given language, without a standard variety indicated. Where these did not differ with regard to the presence vs. absence of ejectives and uvulars, handling such cases was unproblematic, and the first listed dialect was kept while all others were removed from the sample. In cases where different dialects of a language for which no standard variety was indicated differed with regard to the presence or absence of ejectives, all datasets were retained. This was the case only four times, namely for Kawarrang-Ogh Undjan, Western Balochi, Portuguese, and North Junín Quechua. The datasets are distinguished by modifying glottocodes (see Section **Genealogical affiliations and areal breakdown**) by an index (e.g. North Junín Quechua has the glottocode nort2980, and we distinguish the different datasets here as nort2980_01, nort2980_02, and nort2980_03). After pruning the dataset according to these procedures, 2,132 languages remained.

### Altitude data

We retrieve altitude data from a digital altitude model using the *raster* [52] library available in the R programming language [53]. Geo-coordinates for latitude and longitude for each language were extracted from the Glottolog [54]. We passed the coordinates as parameters to the *raster::extract* function, which, given a global digital elevation model, returns altitude figures. We used the GLOBE digital elevation model (https://www.ngdc.noaa.gov/mgg/topo/gltiles.html) openly available from the National Centers for Environmental Information (https://www.ngdc.noaa.gov/ngdc.html). The GLOBE model has global coverage and a spatial resolution of 1km, leaving the file size manageable to work with and the resolution appropriate for investigating languages. We were able to extract altitude for 2,035 languages.

### Genealogical affiliations and areal breakdown

For modelling the effect of altitude on the distribution of ejective and uvular consonants while taking into account the genealogical structure of the world’s languages, we have relied on the Glottolog’s classification [54], which tends to be conservative in accepting proposals for genealogical relations by insisting on documented evidence for form-meaning similarities that are explained in the least costly manner by inheritance from a common ancestor. The dataset underlying Glottolog assigns unique alphanumeric identifiers (so-called “glottocodes”) to both languages and language families, but does not explicitly treat language isolates as singleton language families. For the purpose of our analysis, we have assigned these to pseudo-families called “isolate_1”, “isolate_2” etc. to reflect the fact that they, like language families, represent genealogically independent lineages. Glottolog also retains some data for bookkeeping purposes, which are assigned to a pseudo-family with the glottocode “book1242”; we have removed these entries from the dataset, as Glottolog informs its users for each such entry that it “has been retired and is featured here only for bookkeeping purposes. Either the entry has been replaced with one or more accurate entries or it has been retired because it was based on a misunderstanding to begin with .” See: https://glottolog.org/resource/languoid/id/book1242.

While Glottolog provides a convenient and well-curated genealogical classification for controlling for inheritance, doing the same for contact-induced areality is generally more difficult and also for our present purposes specifically. Standardly accepted language area partitions, such as [29], are also implemented in WALS, and a recent modification is available, too [55]. However, they are not ideal for our present purpose as they divide the world into macroareas that are large enough to contain several salient high-altitude zones and/or hotspots in the distribution of ejectives and/or uvulars. For instance, the Eurasian macroarea, as defined in these partitions, would include the Alps, the Caucasus, the Hindu Kush, and the Himalayas. From a conceptual point of view, using this geographic partition for present purposes would entail an unspoken expectation that these major mountain ranges should behave alike, or can at least be treated analytically as behaving alike, with regard to the distribution of ejectives and uvulars. However, there is in fact no robust reason to assume that simply because they are all located on the Eurasian landmass, this should be the case. We have therefore recognized the need for a more fine-grained partitioning in which high-altitude mountain regions are distributed more evenly across macroareas. As such, in order to retain a link with the extant literature, we have built on a partitioning that is based on Nichols’s [56, pg. 25-26] maximal differentiation of the world into areas which has also been used elsewhere [57] for some analyses. Given that the PHOIBLE coverage is much denser than the language samples used in [56, 57], instructions and criteria to separate areas from one another geographically had to be amended. Additionally, we do not make use of the distinction between Western North America and Eastern North America and we also do not adopt an Ancient Near East area as distinguished in [56] because no languages of the ancient Near East are included in PHOIBLE. Instead, we introduced a new area called “Western Asia” into the partitioning, which is not included in any earlier analysis simply because there were few (or even no) languages from this part of the world in the respective samples. Building on this work, and modifying it to suit the purposes of the present research, we obtain a division of the world into eleven areas. Table 1 lists these areas, together with their conventional bounds from neighboring areas, where these require specification in the second column. The third column provides impressionistic and non-systematic information on some of the major mountain areas within these areas (where existent). As can be seen, the Europe and Northern Eurasian areas as defined here still include unconnected mountain areas. We have decided to not split these areas as distinguished by [56] further because the classes of segments we are interested in actually do not feature in the Alps and the Urals, so that distinguishing further areas around these mountain regions would be virtually or entirely void of the variables of interest.

**Table 1:** The eleven macroareas used in this study to control for large-scale contact-induced areality.

Area	Comments	Major mountain areas
Africa	Delimited by the Isthmus of Suez and including Cape Verde, Madagascar, and the Mascarene Islands.	Ethiopian Highlands
Europe	Includes the Caucasus and delimited from Northern Eurasia by its southern ranges and further north by the Ural mountains. Also includes Malta.	Alps, Caucasus
Northern Eurasia	Includes the Hindu Kush and the Himalayas; specifically, the Pakistani provinces of Gilgit and the Indian provinces of Jammu and Kashmir, Himachal Pradesh, Uttarakhand, Sikkim, Arunachal Pradesh, as well as Nepal and Bhutan as a whole. Beyond, South & Southeast Asia begins. In the southeast, Northern Eurasia is delimited from South & Southeast Asia by the national boundary of China. Also includes Japan.	Urals, Hindu Kush, Himalayas
Western Asia	The Middle East and those parts of Pakistan not assigned to Northern Eurasia.	
South & Southeast Asia	See above, includes islands to the east of New Guinea.	Southeast Asian Massif
Australia		
New Guinea	The island of New Guinea narrowly. Islands to the east were assigned to Oceania.	New Guinea highlands
Oceania	Melanesia, Micronesia (including all Islands at the longitude of New Guinea and further east), and Polynesia.	
North America	Bounded in the south by the US-Mexico border.	Rocky Mountains
Middle America	Bounded in the north by the US-Mexico border and in the south by the Panama-Colombia border.	Central Mexican Highlands
South America	Bounded in the north by the Panama-Colombia border.	Andes

When in doubt as to which macroarea a language should be assigned to (for instance in the case of languages that are spoken both to the north and the south of the US-Mexico border), we have relied on the latitude and longitude coordinates as provided by Glottolog. This, of course, is ultimately arbitrary, but unlike using these coordinates *tout court*, only a very small number of languages are affected. This procedure relieves us from making ad-hoc arbitrary decisions that might bias the outcome by referring to decisions that have been made earlier. As a final note, we have bypassed the difficult decision of assigning Aleut (spoken on the Aleutian islands) and Kalaallisut (spoken on Greenland) to any of the macroareas by leaving them unassigned (a problem Nichols [56] dealt with by not including Eskimo-Aleut languages into the survey in the first place).

## Analyses

### Descriptive statistics

We begin our discussion of the data by providing some basic descriptive statistics. Of the 2,132 languages in our sample, 268, or approximately 13%, feature uvulars that were not annotated as marginal, and 175 languages, or approximately 8%, non-marginal ejectives.

The mean and median altitudes for languages with and without ejectives and uvulars are given in Table 2. Languages with both types of sounds are on average consistently spoken at higher altitudes than those languages that lack them.

**Table 2:** Mean and median of altitudes for languages with and without ejectives and uvulars.

Languages	Mean (MASL)	Median (MASL)
With uvulars	1136	623
Without uvulars	590	306
With ejectives	1237	1136
Without ejectives	606	305

Next we assessed mean altitude of languages with ejectives and uvulars and those without them separately by the macroareas defined in Section **Genealogical affiliations and areal breakdown**. Table 3 provides the mean and median (Mdn) values for the eleven macroareas defined for this study, together with the number of languages contributing to each cell (n).

**Table 3:** Mean altitude of languages with and without ejectives and uvulars for the eleven macroareas defined for this study and the number of languages in each group.

Macroarea	Uvulars			No uvulars			Ejectives			No Ejectives		
	Mean	Mdn	n	Mean	Mdn	n	Mean	Mdn	n	Mean	Mdn	n
Africa	755	513	54	678	500	653	1,474	1,497	61	609	458	646
Europe	873	484	35	342	179	81	1,388	1,445	21	310	173	95
Northern Eurasia	1,756	1,305	59	1,868	1,613	92	1,303	267	4	1,840	1,607	147
Western Asia	875	736	16	1,340	1,141	5	1,182	1,033	4	948	897	17
South & Southeast Asia	722	438	15	476	278	219	n/a	n/a	0	491	286	234
Australia	n/a	n/a	0	191	134	312	n/a	n/a	0	191	134	312
New Guinea	2,184	2,184	1	645	305	75	n/a	n/a	0	666	305	76
Oceania	n/a	n/a	0	300	131	38	n/a	n/a	0	300	131	38
North America	627	457	35	744	525	56	759	450	46	645	553	45
Middle America	1,035	434	13	995	849	37	1,219	1,375	15	914	624	35
South America	1,777	1,036	38	442	204	296	1,459	400	24	528	207	310

First, Table 3 reveals some properties of the dataset that are worth bearing in mind. In some of the macroareas, such as Australia and Oceania, both uvulars and ejective consonants are completely absent (see also Figs 1 and 2). In addition, New Guinea and South and Southeast Asia host a very small number of languages with uvulars; none have ejectives (again see Figs 1 and 2). It is interesting to note, incidentally, that the implicated macroregions are not randomly distributed but they jointly identify a significant contiguous subpart of the world in which these sounds are rare or absent.

Where ejectives and uvulars occur more frequently, it is almost always the case that languages with ejectives and uvulars are spoken in regions with a higher mean altitude than those that do not, although the difference is minimal in some cases. The major anomaly is Northern Eurasia, where languages without ejectives and uvulars obtain higher mean values than those with them. Going against the general trend are also Western Asia and North America, though with regard to uvulars only.

By and large, the descriptive summary statistics largely replicate the results obtained by Everett [21] for ejectives. Furthermore, the generalization that languages with ejectives are on average spoken at higher altitudes than languages without them holds better than the analogous generalization for uvulars (which is violated also in North America and Western Asia). This might suggest that the relationship between ejectives and altitude is stronger than that between uvulars and altitude.

**Fig 3:**
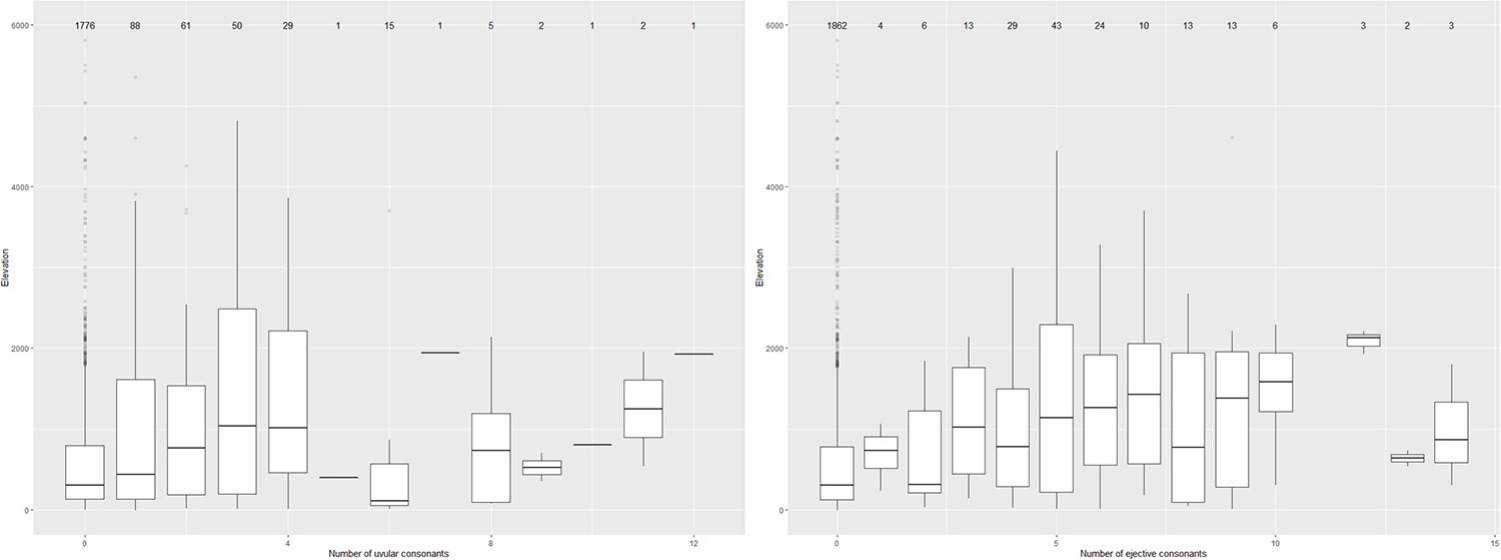
Number of uvular consonants (left) and ejective consonants (right) in the PHOIBLE database as modified for this study.

Furthermore, the plots in Fig 3 show the number of ejectives and uvulars consonants in the languages in the PHOIBLE database as modified for this study depending on altitude. As one can see from the labels of the boxes, languages with many such segments are generally rare (and note that the plots do not show languages with more than 15 such segments as these are exceedingly rare). Under both explanatory accounts mentioned in Section **Altitude and the distributional typology of language structure: ejectives and beyond**, one might indeed hypothesize that altitude should not only have an effect on whether ejectives and uvulars are present or absent in a language, but also on the number of distinct segments of both classes—with languages spoken at higher altitudes enriching their segment inventories with more sounds of both classes than languages spoken at lower altitudes. Although we will not follow up these informal first observations in more detail in this article, the plots suggest that there might be a mild tendency for the number of both classes of sounds that are found in the languages of the world to increase with altitude, too.

However, also for the sheer presence or absence of ejectives and uvulars, and the possible influence of altitude, it is necessary to analyze the situation in much more detail and from different angles to assess the robustness of this first impression. For further analysis beyond descriptive statistics, here we combine a rigorous quantitative statistical and computational phylogenetic approach on the basis of the large-scale comparative data from PHOIBLE (as recently exemplified in [20, 49, 50]) with a more qualitative intra-family analysis on the distribution and diachrony of relevant sound classes (as exemplified recently in [41]). This approach is motivated by the conviction that such a combination of quantitative and qualitative perspectives allow for deeper insights into the analyzed phenomena than one of them alone.

### Modelling the presence of ejectives and uvulars as a function of altitude

We first approach the question if the probability of observing languages with ejectives and uvulars in the PHOIBLE data (as modified according to the procedures described in Section **Altitude data**) is greater at higher altitudes by a global overall analysis of all available data. For this global analysis, we employ Bayesian Logistic Mixed Effects Regression as implemented in the R package *brms* [58–60], which uses the Stan programming language’s interface with the R statistical programming environment [61]. We have included altitude as computed according to the procedure described in Section **Altitude data** as a fixed effect after applying a log10-transformation. This is because of an extreme left skew in the distribution of the altitudes at which languages are spoken—as human populations are typically much denser at low altitudes [28], so is apparently language density. Note that because of the transformation, four further languages could not be taken into account for modelling, leaving a set of 2,031 languages for analysis.

Specifically in the context of research on linguistic adaptation to environmental givens, it is important to control within the same analysis for possible confounds due to genealogical “vertical” dependencies (language relatedness) and areal “horizonzal” dependencies (due to prolonged coexistence in neighboring areas and interactions of speakers that led to contact-induced similarities). Performing separate analyses to address their possible effects can lead to conclusions that may be unwarranted [46]. We have therefore included both genealogical affiliation and geographical location as captured by the eleven macroareas as random effects into our mixed effects model and fitted random intercepts for both variables. Since it is conceivable that, depending on area, contact patterns in different mountainous regions (on which see the survey in [14]) have differing effects on the distribution of ejectives and uvulars, we considered it advisable to also fit random slopes for the macroareas that we are using to control for contact-induced areality. We did not include random slopes for the genealogical structure of the languages in our sample because of the impossibility to fit these given the large number of small language families and isolates [62, pg. 298-299]. However, since this data structure is also of potential concern for random intercepts, we later provide intra-family and intra-area assessments of variation in the presence of ejectives and uvulars to compare these with the results of the model. We have placed a weakly informative prior of SD = 2 on the fixed effect to be conservative and to not constrain the model too tightly (even though with large amounts of data the prior should not affect the posterior distribution significantly [63, pg. 149-150] and otherwise used default priors for the standard deviation of random effects and residual errors, which are constrained to positive values [63, pg. 150]. We ran the models in four chains, with 6,000 warm-ups and 8,000 iterations; each with relatively high values to ensure reliable estimates. To ensure convergence, we furthermore set the drift parameter delta to.999 and increased the maximum tree-depth to 20.

$\hat{\text{R}}$ values of 1 for each parameter, effective sample size estimates, and a visual inspection of the chains indicated that both models converged, and comparisons of plots of observed data with posterior predictive samples showed that the models fit the data well. In addition, we have assessed the predictive accuracy of both models as suggested in [64], which was high in both cases (approximately 84% for uvulars and 92% for ejectives). More specifically and relevantly, the models were also good at predicting which languages in the sample do have sounds of both classes (approximately 84% accuracy for uvulars and 94% for ejectives). The main result of the modelling is that increasing altitude by a factor of 10 increases the log-odds of observing a uvular by 0.16 with a 95% credible interval of [-0.32, 0.80]. Since this interval includes zero, the effect is not credible. A reviewer asked whether the absence of a credible effect might be due to the inclusion of uvular rhotics (widespread e.g. in European languages as a result of language contact [65]), theorizing that plosives may be more sensitive to altitude differences than rhotics. Although a principled account for why this should be so is not available, this suggestion converged with our own intuitions. We therefore decided to run a third model, this time excluding uvular rhotics /ʀ/ and /ʁ/ (see [66, pg. 215-217] for justification). The results of this ancillary model were not significantly different from the one that takes uvular rhotics into account: the size of the effect, 0.35 (95% CI [-0.21 1.02]), was only mildly stronger and still included zero in the confidence interval as predictive accuracy increased equally slightly to 85%. Increasing altitude by a factor of 10 increases the log-odds of observing an ejective by 1.57, i.e., more so than for observing a uvular. The credible interval of [0.15, 2.93] does not include zero, suggesting a mild effect of altitude on the distribution of ejectives. The posterior probability of the result being chance was relatively high for uvulars (p = .2655; p = .099625 for the ancillary model excluding rhotics) and relatively speaking lower for ejectives (p = .018). In sum, Bayesian Mixed Effect Logistic Regression supports no strong effect of altitude on the distribution of uvulars and only a mild one on the distribution of ejectives.

**Fig 4:**
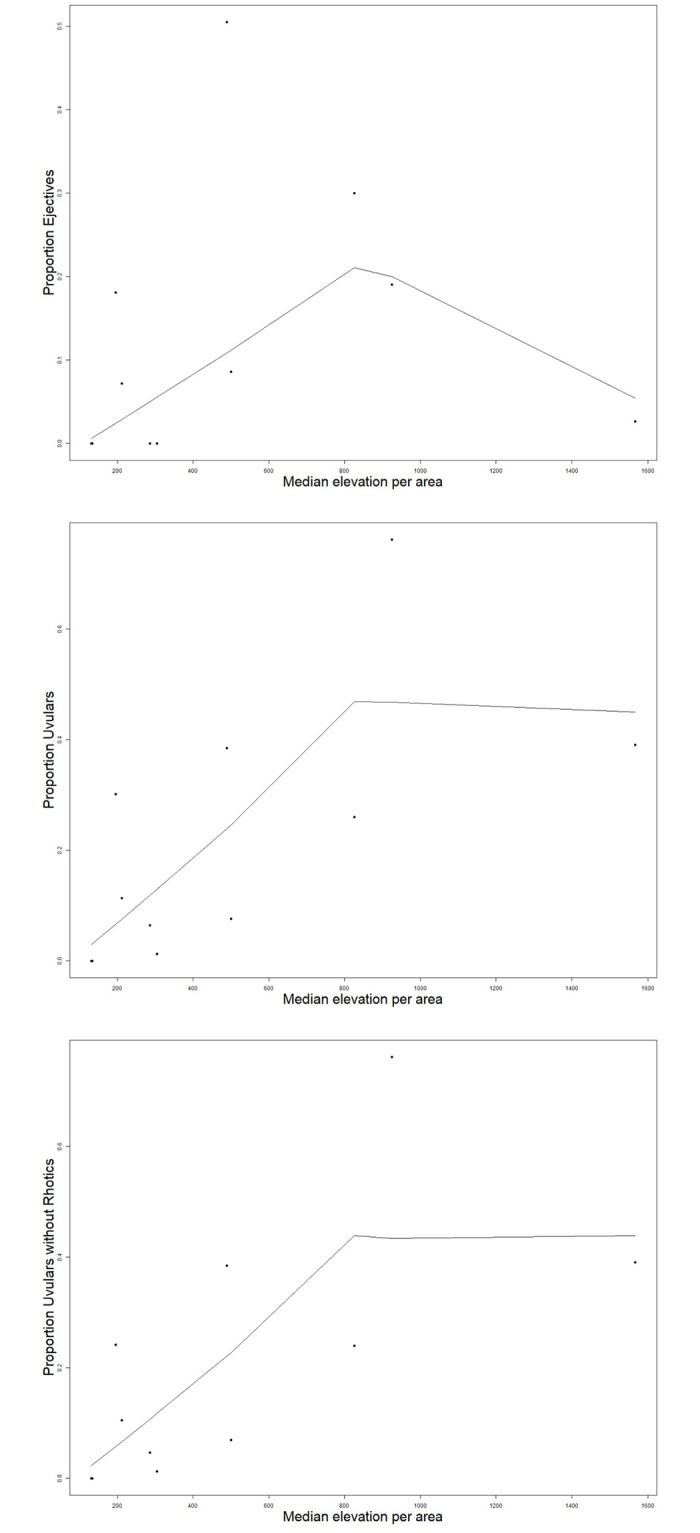
Plots of intra-area and intra-family means for large language families with uvulars against altitude means; lines represent Lowess scatterplot smoothers.

**Fig 5:**
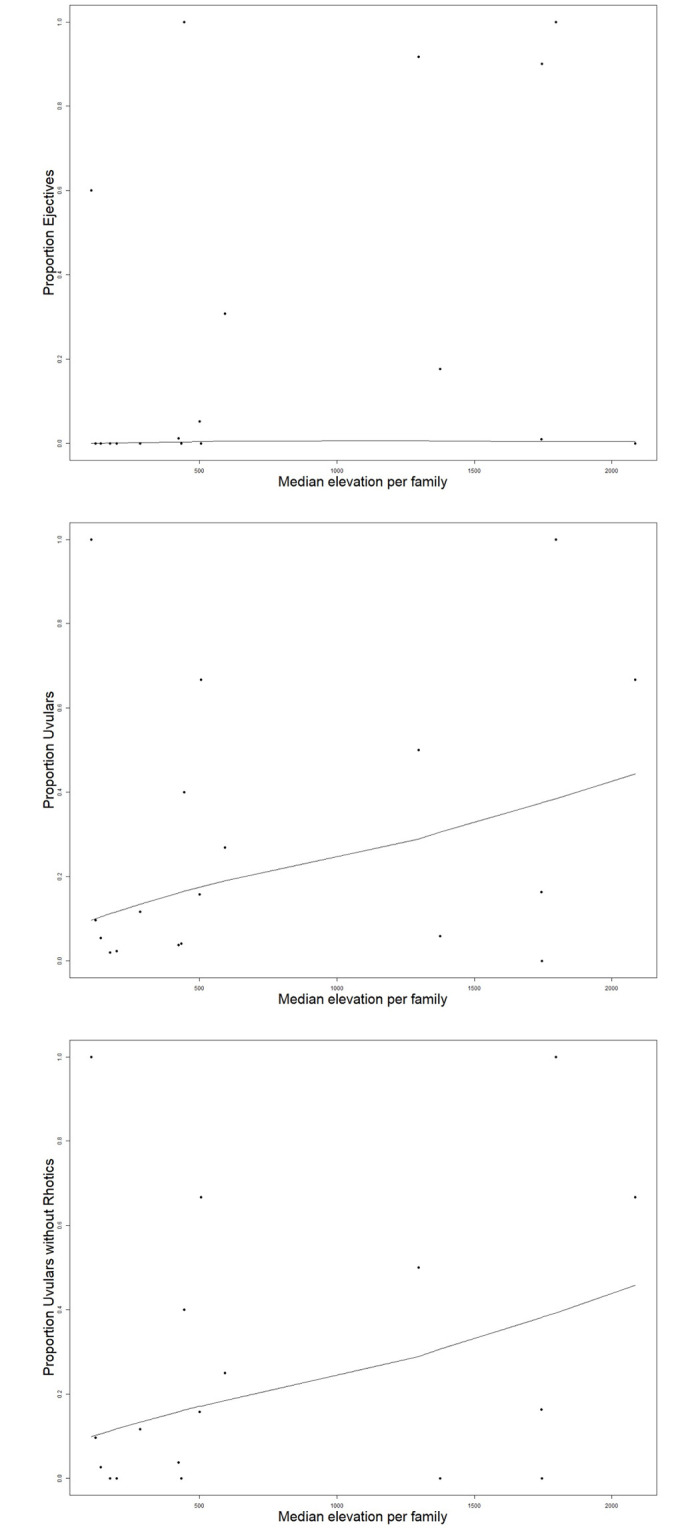
Plots of intra-area and intra-family means for large language families with ejectives against altitude means; lines represent Lowess scatterplot smoothers.

As alluded to, the large number of isolates and small language families of the world, which are also present in PHOIBLE, is a concern for fitting random effects structures. We have therefore, in addition to the Bayesian Logistic Mixed Effects Regression, performed least squares regressions using the median of altitude and ejectives and uvulars proportions within macroareas and within language families. This additional analysis can be thought of as an analogue, in language typology, to a by-subjects and by-items treatment in psycholinguistic experiments [9, pg. 8]. We chose to use median rather than mean values because median values are more robust and less sensitive to outliers in the distribution. For this additional analysis, we used the same macroarea breakdown described in Section **Genealogical affiliations and areal breakdown** that is also included in the main model. For the intra-family analysis, we focused on language families that were represented in the PHOIBLE database by ten or more languages and in which either uvulars, ejectives, or both were actually attested. These are Afroasiatic, Arawakan, Athabaskan-Eyak-Tlingit, Atlantic-Congo, Austronesian, Cariban, Dravidian, Indo-European, Mande, Mayan, Mongolic, Nakh-Daghestanian, Otomanguean, Quechuan, Salishan, Sino-Tibetan, Ta-Ne-Omotic, Tai-Kadai, Tupian, Turkic, Uralic, and Uto-Aztecan. Intra-area and intra-family means are plotted against altitude means in Figs 4 and 5.

Results of least square regressions were similar to the Bayesian models in that they have positive, but vanishingly small positive slopes. Moreover, with the only exception being the by-area treatment of uvulars which yielded a significant result (p = 0.037, Adjusted R^2^ = 0.3327; p = 0.0294 Adjusted R^2^ = 0.3628 with rhotics removed), all analyses were insignificant and R^2^ values very low, indicating that the fit to the data is poor. Generally, results for ejectives were not notably better than for uvulars.

### Exploring the diachronic dynamics of ejectives and uvulars in language families

When modelling the distributions as in our analysis, we factor out genealogical information in order to abstract away from the vicissitudes of the phylogenetic histories of individual families. As Cathcart [67, pg. 1] points out, this does not make maximal use of the data because the evolutionary history of the traits of interest, which may allow for important insights into the genesis of the synchronically observable patterns, are artificially discarded. For instance, to decide whether any of the two alternative accounts for the observed distributions that we explore has merit, it is of significant interest whether ejectives and/or uvulars tend to develop within language families in high-altitude environments—or if they are retained there, but tend to be lost elsewhere (i.e. whether they are recessive [68], as argued in [41] for retroflex affricatives). These diachronic perspectives are at the heart of recent research in distributional typology [69, 70]. To assess the diachronic dynamics of both classes of sounds within language families, we chose to look at Indo-European and Sino-Tibetan—two large and old language families of Eurasia, for which there exist high-resolution computationally-generated language phylogenies. Members of these families are spoken across a multitude of different ecozones and altitudes, and given that in both cases several millennia have passed since their breakup from a common ancestor, there should have been ample opportunities for environmental effects on the structures of daughter languages to play out, if they exist.

Since we have reduced the PHOIBLE data to binary variables that simply register the presence vs. absence of ejectives and uvulars, we require a discrete variable model for estimating transition rates on a phylogeny. We have used extant phylogenies for Indo-European and Sino-Tibetan [71, 72], respectively, and available from D-PLACE [73]. We have pruned these phylogenies to retain only those daughter languages that are represented in our dataset from PHOIBLE. This results in initial phylogenies with 58 tips (daughter languages) for Indo-European and 39 for Sino-Tibetan. In order to increase the coverage beyond the phonological inventories in PHOIBLE, we contacted Harald Hammarström to search the DReaM corpus of digitized grammars [74] for the presence of ejectives and uvulars. A simple keyword-spotting technique [75] identifies those languages whose descriptions mention the terms ‘ejective(s)’ and ‘uvular(s)’ with significant frequency to assume that the languages in question exhibit them. First we compared the results of this data mining technique with what we observe in PHOIBLE and precision was over 90%. Given this high level of accuracy, we extended our dataset for the presence vs. absence of ejectives and uvulars to the full set of grammatical descriptions available from the Glottolog for these two language families and we extended our language sample coverage to 75 languages in Indo-European and 72 in Sino-Tibetan. We hand checked any discrepancies and corrected them.

**Fig 6:**
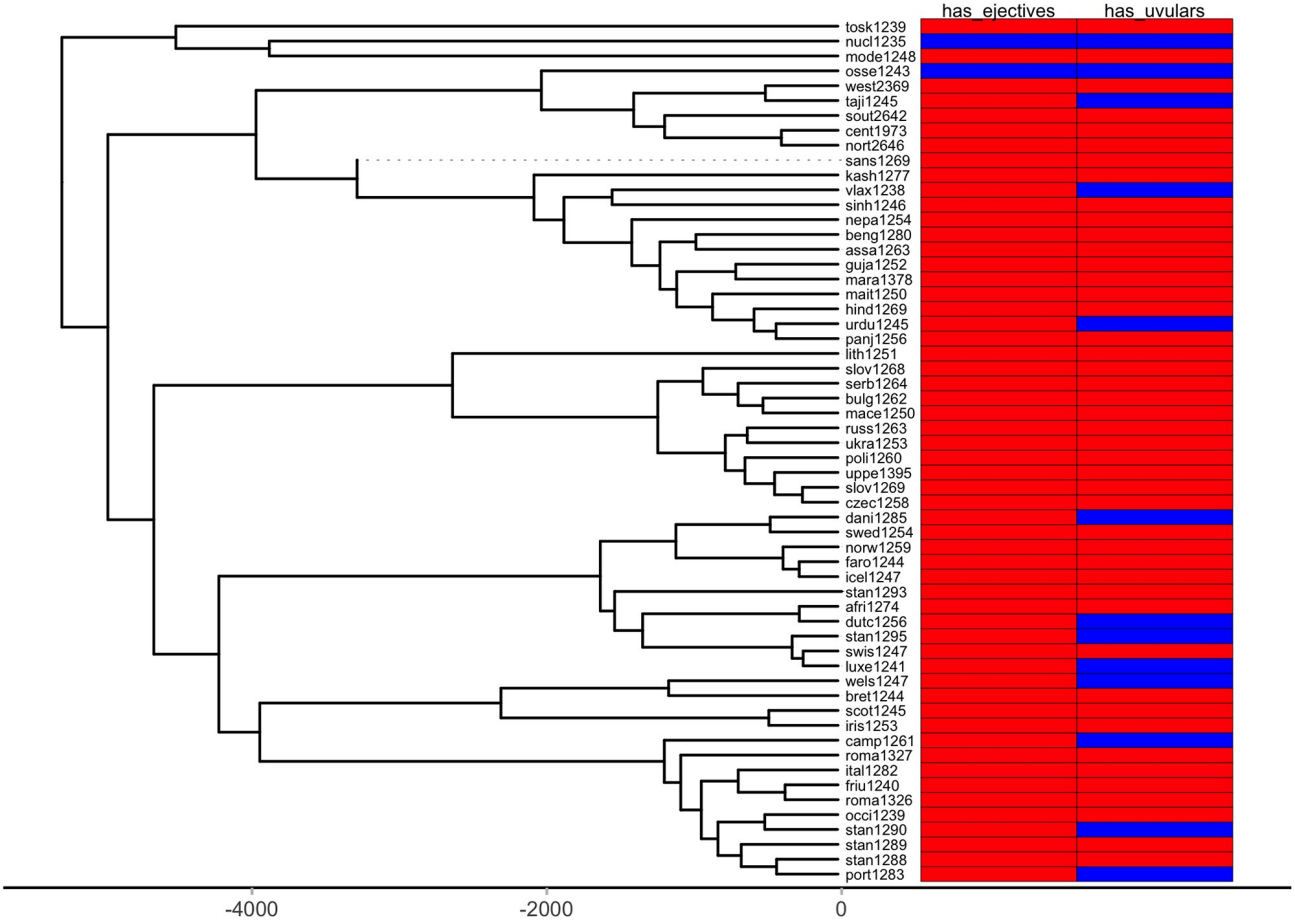
Presence or absence of ejectives and uvulars in extant Indo-European languages for the PHOIBLE data alone.

**Fig 7:**
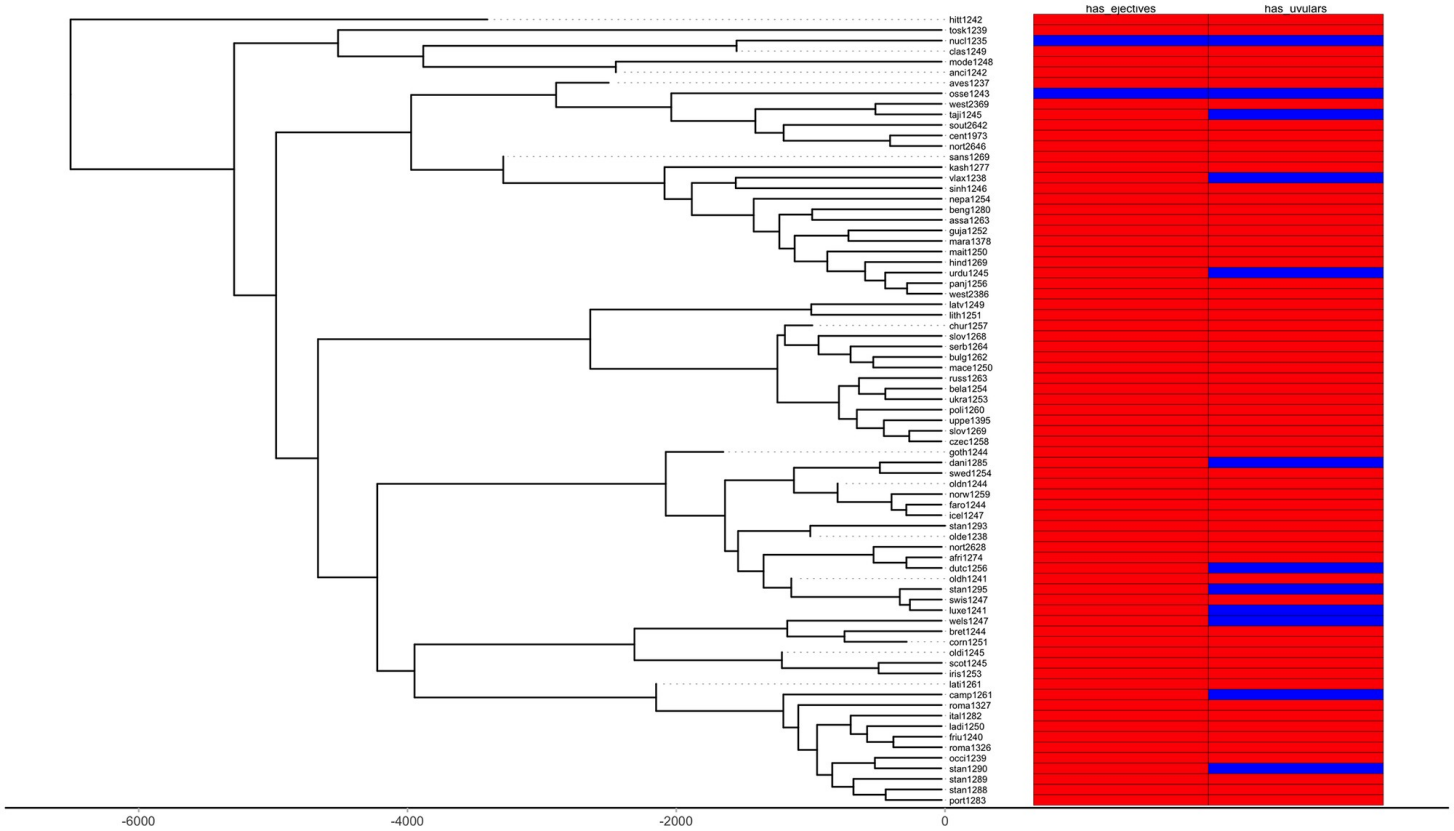
Presence or absence of ejectives and uvulars in extant Indo-European languages for the amended data by means of grammar mining.

**Fig 8:**
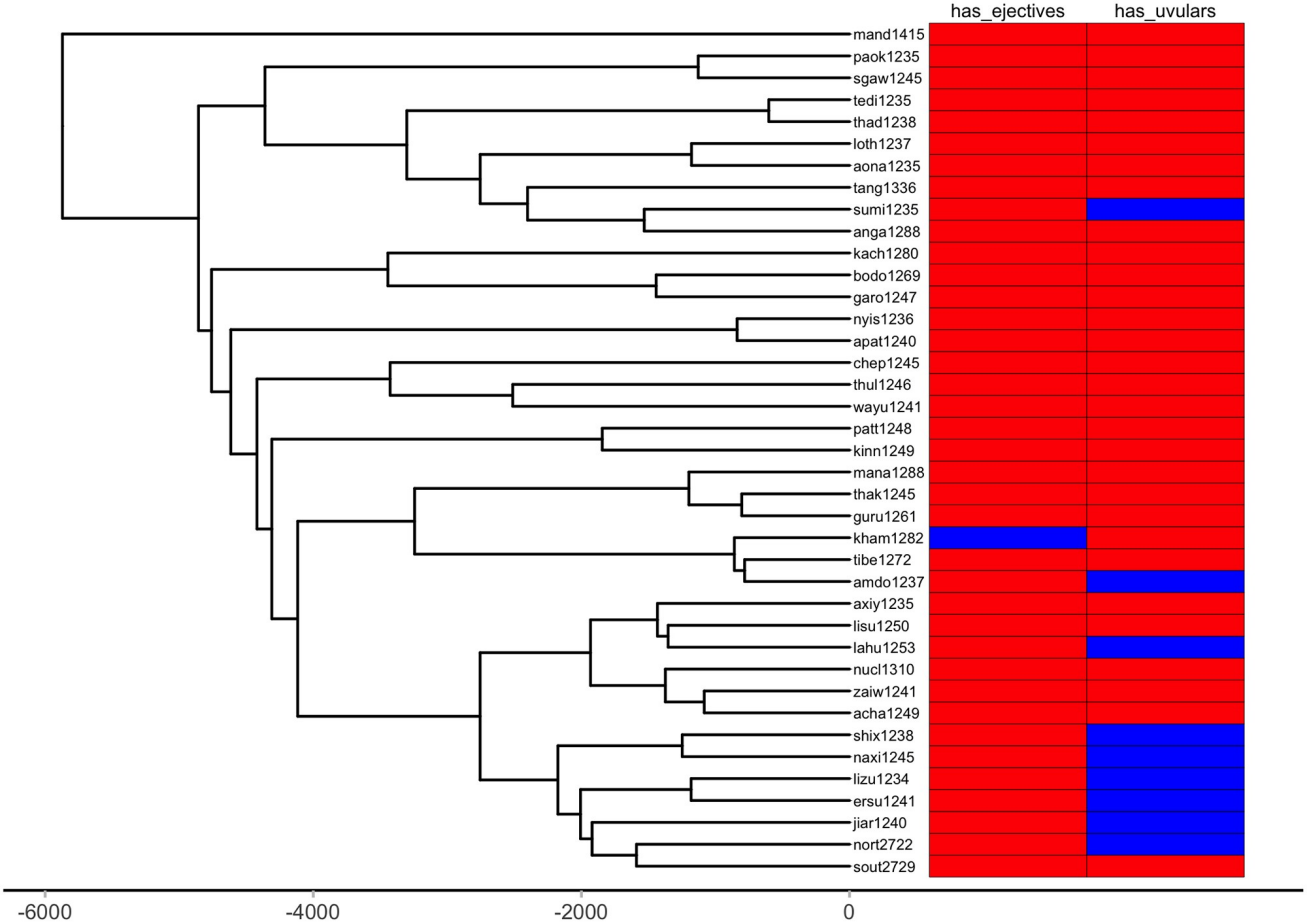
Presence or absence of ejectives and uvulars in extant Sino-Tibetan languages for the PHOIBLE data alone.

**Fig 9:**
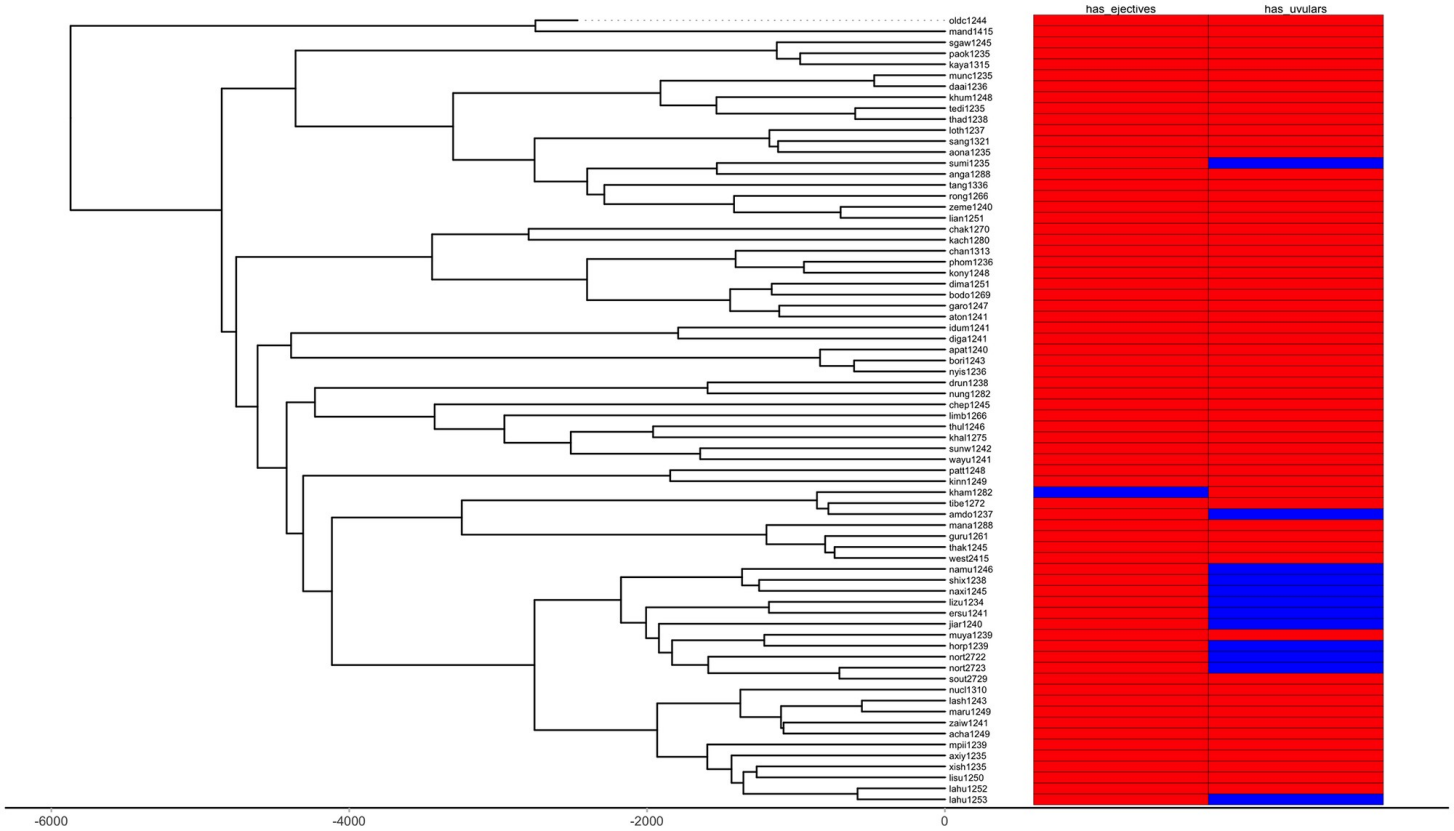
Presence or absence of ejectives and uvulars in extant Sino-Tibetan languages for the amended data by means of grammar mining.

First, we plot each phylogeny and the presence or absence of ejectives and uvulars, as shown in Figs 6 and 7 for Indo-European and Figs 8 and 9 for Sino-Tibetan, respectively, using the R package *ggtree* [76]. Ejectives are exceedingly rare in both language families in our language sample. In the subset of Sino-Tibetan languages that we analyze here, they are only found in Khams Tibetan. In the Indo-European languages we analyze, they are restricted to Ossetic and Eastern Armenian. Uvular consonants are somewhat more frequent in both families and approximately in-line with the cross-linguistic mean proportion in Europe and Northern Eurasia (circa 30% and 39% respectively). Note, however, that they are distributed widely across the phylogenies of both families, and also that manner of articulation varies; for instance, the uvular in European languages is typically rhotic. A velar/uvular contrast particularly in stops, however, is a “macroareal feature extending from North Africa over southwestern Asia to large parts of (especially middle and southern) Central Asia and—discontinuously—some parts of north Asia (northwestern and northeastern Siberia) and even Southeast Asia (Khmer)” [77, pg. 254].

Next, we generated stochastic character maps [78–80] using the make.simmap function from the R package *phytools* [80] for an all rates different (ARD) model (allowing for a larger range of rates to be explored in the MCMC), where q is set to empirical (maximum probability, full Bayesian MCMC) with 10 simulations.

As shown in Figs 10–17, there is low probability of either trait at the root node of each phylogeny, though there is an interesting signal regarding uvulars in Sino-Tibetan which appear to have been innovated at one point in a subgroup and then passed down to several present-day Sino-Tibetan languages.

**Fig 10:**
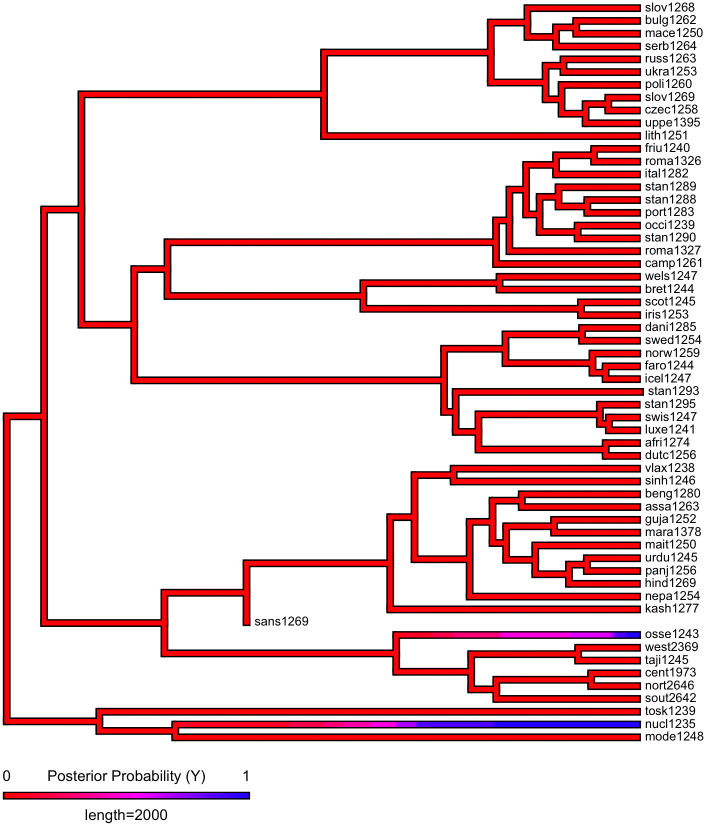
Estimated probability of ejectives as inferred by stochastic character mapping in Indo-European for the PHOIBLE data alone.

**Fig 11:**
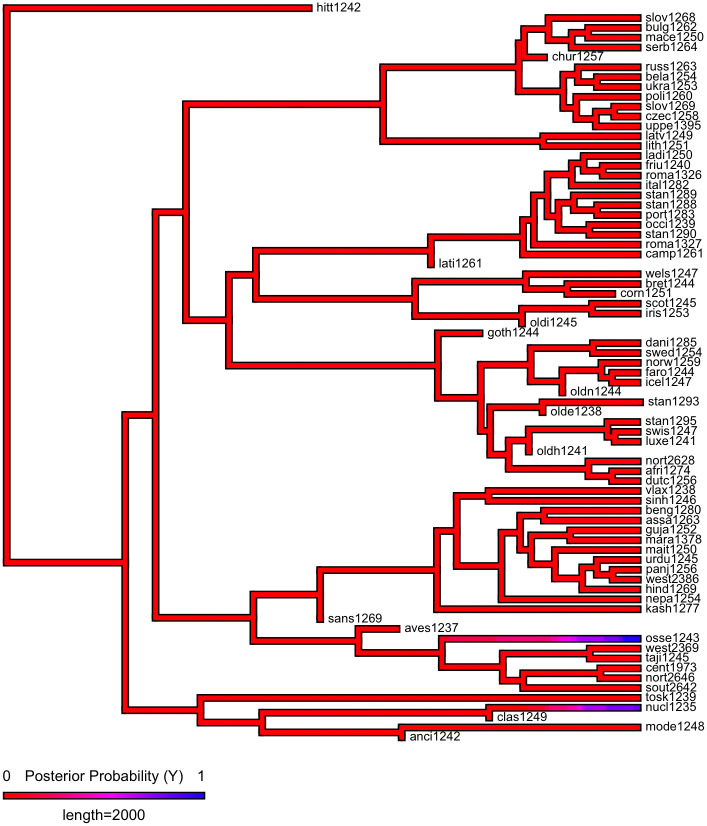
Estimated probability of ejectives as inferred by stochastic character mapping in Indo-European for the amended data by means of grammar mining.

**Fig 12:**
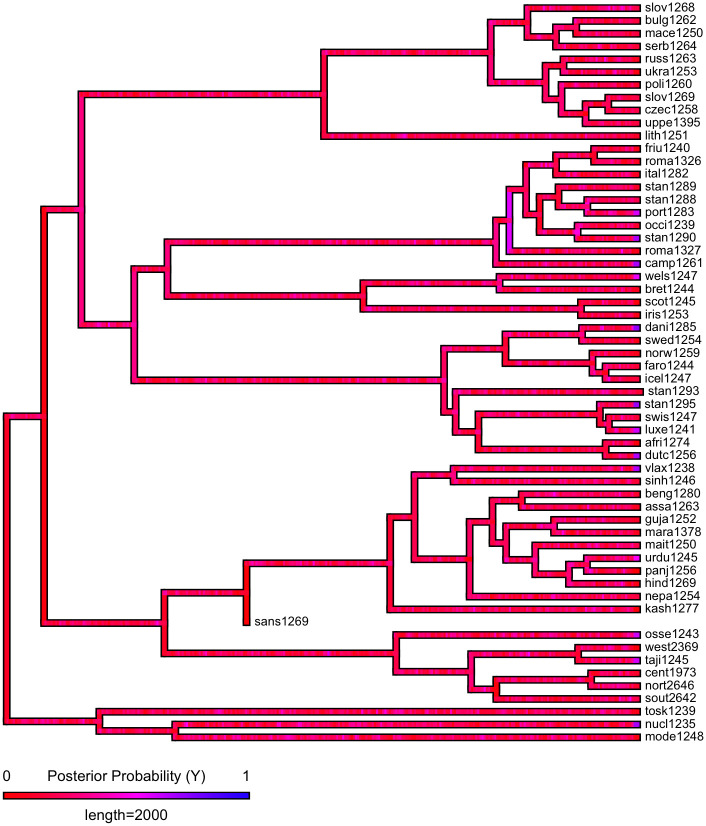
Estimated probability of uvulars as inferred by stochastic character mapping in Indo-European for the PHOIBLE data alone.

**Fig 13:**
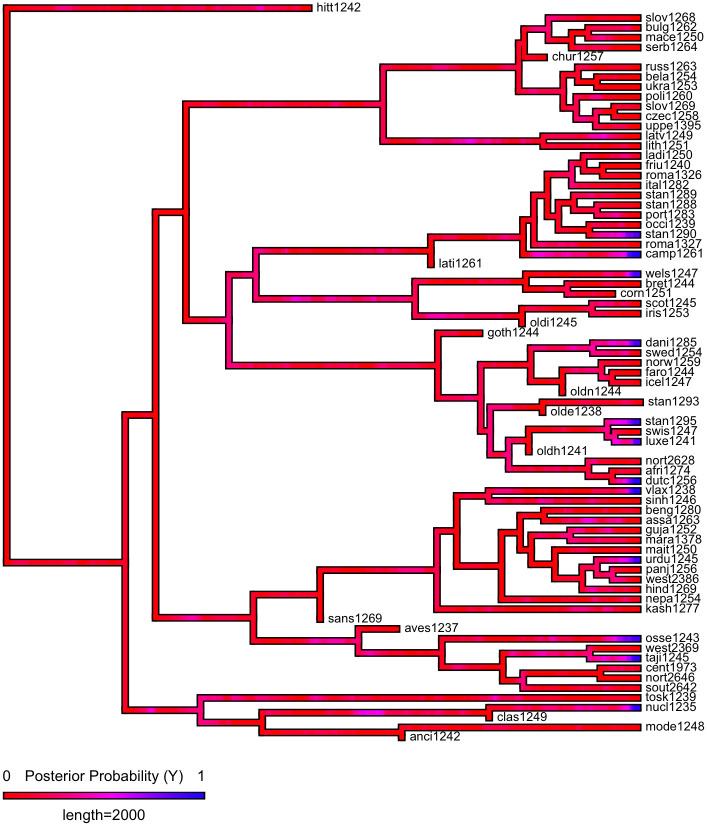
Estimated probability of uvulars as inferred by stochastic character mapping in Indo-European for the amended data by means of grammar mining.

**Fig 14:**
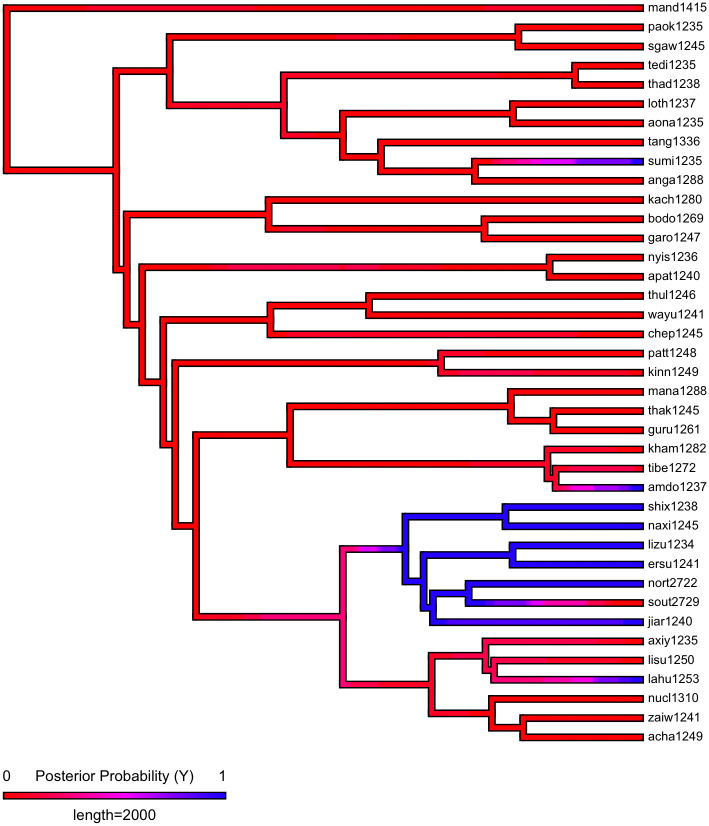
Estimated probability of ejectives as inferred by stochastic character mapping in Sino-Tibetan for the PHOIBLE data alone.

**Fig 15:**
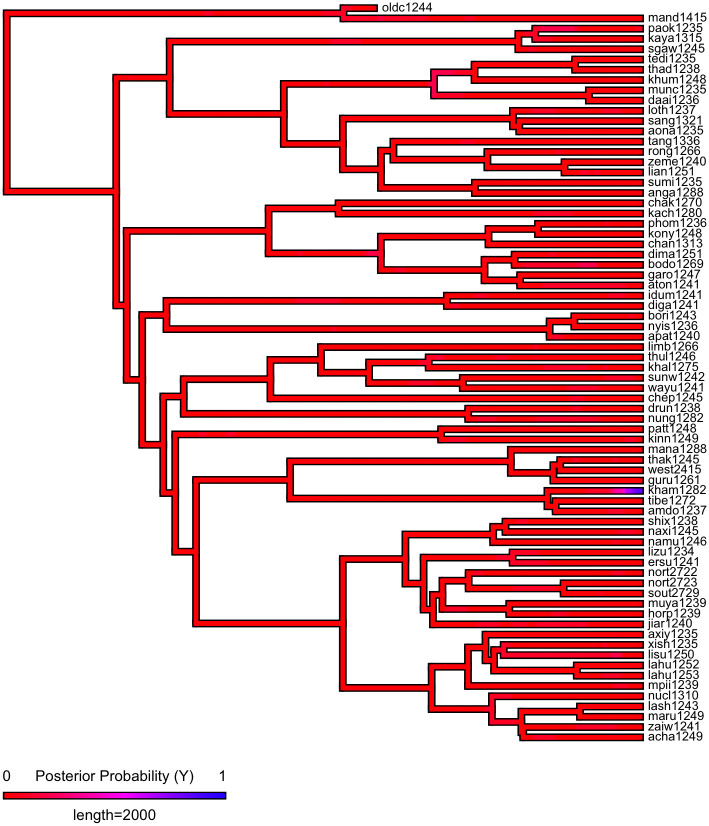
Estimated probability of ejectives as inferred by stochastic character mapping in Sino-Tibetan for the amended data by means of grammar mining.

**Fig 16:**
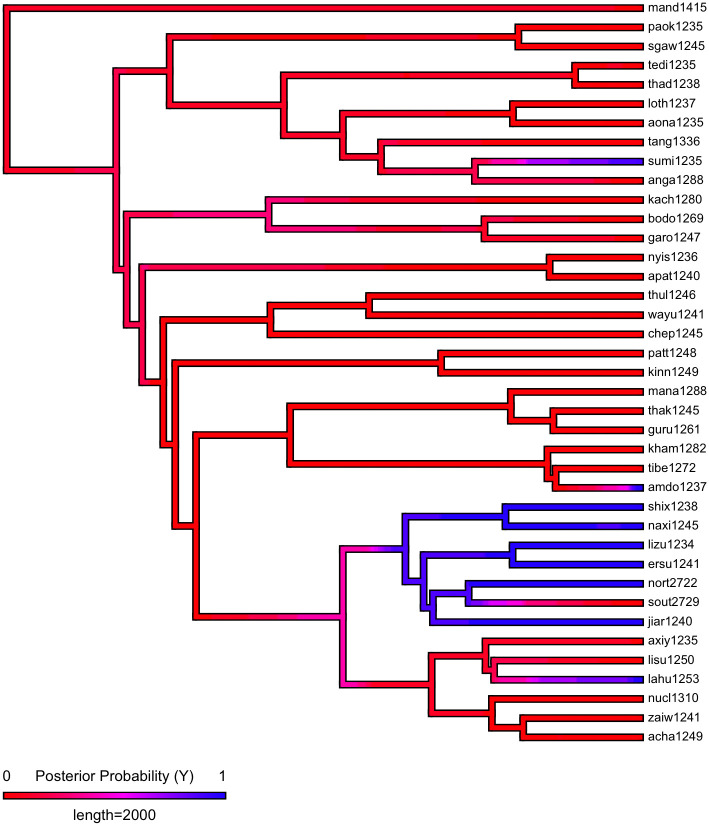
Estimated probability of uvulars as inferred by stochastic character mapping in Sino-Tibetan for the PHOIBLE data alone.

**Fig 17:**
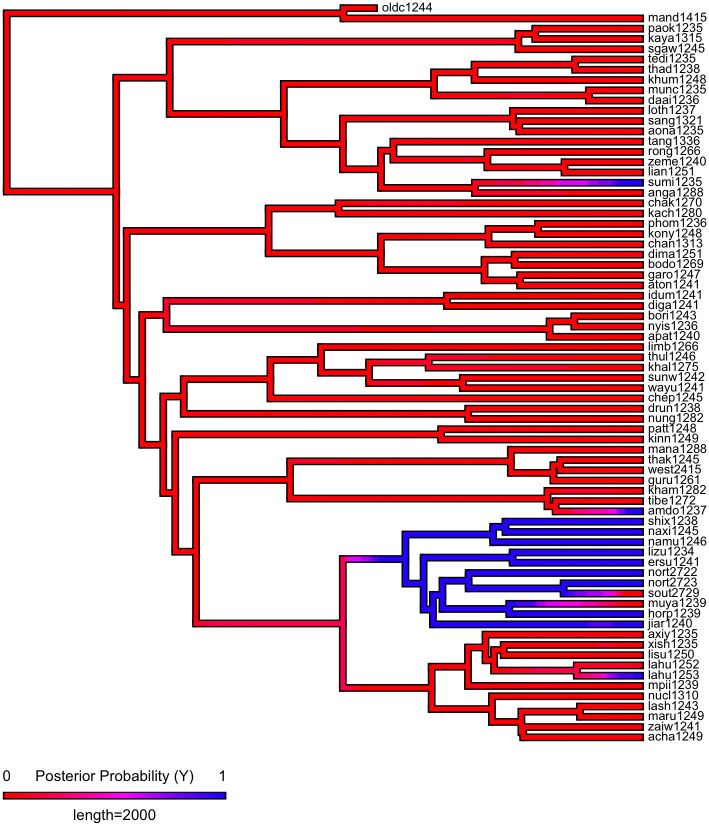
Estimated probability of uvulars as inferred by stochastic character mapping in Sino-Tibetan for the amended data by means of grammar mining.

Next, we proceed by providing an interpretation of the phylogenies, taking into account also the pertinent published literature on the diachrony of the involved languages and language families.

As far as ejectives in Indo-European languages are concerned, their genesis is fairly well understood. The diachronic development of ejectives in Ossetic is usually attributed to contact with neighboring Nakh-Daghestanian languages of the Caucasus, which are rich in ejectives. In Ossetic, direct loans from Nakh-Daghestanian account for the ejectives in most languages’ lexical items [81], but there is also regular sound change that gives rise to them and that likewise can plausibly be theorized to have been triggered by Nakh-Daghestanian influence [82–84]. Note too, that recent work by Eisen [85], who investigates segment borrowing patterns in a large sample of languages [86], suggests the implicational universal that if a language borrows an ejective, then it already has at least one. As far as we can ascertain, the majority opinion on ejectives in Armenian is that they are contact-induced, too, though there is an alternative theory that holds that they are instead directly inherited from Proto-Indo-European, under the assumption of some version of Glottalic Theory [87–89]. Interestingly, however, proponents of this alternative view, too, argue that contact actually is involved: instead of inducing the genesis of ejectives in Eastern Armenian, as was the case for Ossetic, in this case it is rather argued that their proximity “favored the preservation of a feature which was already present” [90, pg. 190]. Whatever took place, it was in a relatively high-altitude environment in the Caucasus.

Since ejectives are not a significant factor in Sino-Tibetan languages, we move on to a discussion of uvulars, again starting with Indo-European. It is remarkable that exactly Ossetic and Armenian figure among the Indo-European languages with this type of sound. These languages, thus, do not only replicate one particular class of sounds of Caucasian languages, but rather have similar consonant inventories to Caucasian languages more generally, as Caucasian languages are saliently characterized by rich systems of both ejectives and uvulars. It is widely recognized that both ejectives and uvulars are areal features of the Caucasus [91–93]. However, the wider proliferation of uvulars in Northern Eurasia has led some to question the possibility of using these as area-defining features ([94]; see also [95]). Nevertheless, the uvular inventories of Caucasian languages are usually larger, by far, than those of other European languages [93, pg. 47-48] (as is typically the case with the phonological inventories in general, which in fact might make it more probable to observe typologically rare sounds like ejectives and uvulars in the first place). The decision whether uvulars in the Caucasus can or cannot be considered a contact-induced areal feature is a matter for specialists to decide. Here, we wish to note that also in the mountains of the Hindu Kush, there are higher than expected frequencies of uvulars in Indo-European languages, when compared with the cross-linguistic average as represented in WALS [43, 96]. Contact with the prestigious Persian language, which has a uvular, is a relevant factor for the occurrence of uvulars in Indo-Aryan languages of the Hindu Kush, where they occur in Perso-Arabic loanwords as a “prestige pronunciation” [96, pg. 121]. Indeed in some but not all Indo-Aryan languages, uvulars have a somewhat marginal status. Note also that in other Indo-Aryan languages, the contrast between velar and uvular stops is not always strongly established [77, pg. 253]. However, as is the case with ejectives in Ossetic, they are more deeply entrenched in the phonological inventories of languages from the northern part of the Hindu Kush, where they are also found in native vocabulary [96, pg. 121]. Tracing the history further back, the Persian uvular itself, while having internal sources, earlier received a major “boost” in frequency and functional load through the massive influx of Arabic loanwords [97]. Here, we can trace aspects of the genesis of the areal-typological uvular belt from northwest Africa to Central Asia, which Tikkanen [77] ascribes to individual language contact events. Uvular consonants, especially rhotics, however, are also prominent in many languages of Europe. Again, the history of this phenomenon is contact-induced: uvular articulation of the rhotic originated in the Parisian dialect of French in the 17th century, and, as is the case for the Hindu Kush, spread as a prestige phenomenon from there; first to the metropolitan centers of other countries (and thereby across language boundaries) and from there further to more rural areas [98].

Of course this literature review is selective, and there are cases including Turkic and Mongolic, where uvulars arose apparently entirely from internal sources through the phonologization of an originally allophonic variation in the context of back vowels that still persists in some languages [77]. However, the present-day distribution of uvulars is strongly influenced by local areal factors, and language contact seems to have played a significant role in its genesis. For example, Matras [99, pg. 270] considers a uvular stop as an areal feature of present-day Anatolia.

Finally, we explored the apparent phylogenetic signal in the diachronic development of uvulars in Sino-Tibetan, in particular with a view to assessing whether it may have something to do with the environment in which its diachronic development took place (one article which we unfortunately could not take into account in our evaluation is [100]). In PHOIBLE, uvular consonants are found in various places of the Sino-Tibetan phylogeny, and we will not trace their history within the entire family here in full detail. In a nutshell, it seems that uvular sounds can be posited at the root of the proto-language, but that they merged with other sounds in different branches at various points of time. For instance, Old Chinese still retained uvulars, but modern Chinese has lost them [101, pg. 32-33, 45]. As far as the PHOIBLE data are concerned, however, there are two highland subgroups of Sino-Tibetan languages where they cluster together densely. These are Naic and Qiangic, which are hypothesized to form a common Na-Qiangic node together with Ersuic, where uvulars are also found [102, pg. 13-14]. Note however, that speculation is involved [102] and even Qiangic itself is not universally accepted [103]. Uvulars are, however, reconstructed for Proto-Naish (a subgroup of Naic [104, pg. 492]), and they have also been used to characterize Qiangic [103, pg. 137] and might reconstruct to Proto-Qiangic under the assumption that the group is valid [101, pg. 124]. However, they are also found outside Qiangic, including “in a number of Tibetan dialects spoken in the zone of distribution of Qiangic languages”, i.e. Eastern Tibet and adjacent parts of Sichuan and Yunnan [103, pg. 147]. The pruned phylogeny for our phylogenetic study does not reflect that well because the Tibetan evidence is mostly removed from the phylogenetic analysis as relevant Tibetan languages are not included in the phylogeny we adopt [72]. Similar observations can be made regarding the geographical distribution of uvulars in Tibetan, in that also other Sino-Tibetan languages in this region, as well as the Mongolian language Monguor, have uvulars: “[t]he region can be regarded as a uvular prone Sprachbund” [101, pg. 124]. Tentatively, Hill states that uvulars could have emerged in the Tibetan and Mongolian languages of the region due to a Qiangic substrate, given that the class of sounds seems to be more well-entrenched in this group of languages. In sum, there is a phylogenetic signal in that several authors suggest the reconstructability of uvulars to low-level ancestors of local Sino-Tibetan subgroups. However, the pattern of contact-induced emergence of uvulars that we have observed repeatedly elsewhere resurfaces at least equally prominently, albeit here possibly by a sub- rather than super-stratum effect [101].

Even though it seems clear for some cases surveyed here that contact was the main driver for the diachronic development of ejectives and uvulars, could this perhaps be only the proximate reason? And could the ultimate reason why they were replicated through contact be an adaptation to high-altitude environments such as those of the Northern Caucasus and Eastern Tibet through language contact effects? The extant literature is often Janus-faced when it comes to this question [14]. Where there is clear evidence for contact as the factor that generated the spread of phonological phenomena across language and language family borders, this is swiftly integrated into an account based on adaptation to environmental conditions by stating that relevant features may have spread through contact precisely because they are adaptive (e.g. [105, pg. 86] in response to [47]). However, note that Eastern Tibet, where some amount of convergence regarding uvulars seems to have taken place, is notably lower in altitude than Western Tibet and has a markedly different climate and vegetation. One would accordingly have expected that convergence would rather take place at the highest altitudes, if altitude were the driving factor. However, the language dynamics of altiplanos, such as the Tibetan Plateau, are usually different from mountain areas with a central crest [106]. Therefore, the account that operates with the assumption that higher altitude induces sociolinguistic isolation does not necessarily apply straightforwardly to these cases. More generally, we observe the spread of uvulars at both high and low altitudes (i.e. in Europe), which is another reason for caution before adding another explanatory layer behind the contact-induced account (cf. [32] for similar reasoning). In sum, where ejectives and uvulars were innovated through contact, rather than sociolinguistic isolation, seems to be the decisive factor.

## Discussion

In this contribution, we have examined the cross-linguistic distribution of two classes of sounds, ejectives and uvulars. We have sought to establish to what extent this distribution is predicted by environmental factors, concretely, the altitude of the area in which these languages are spoken. Our analyses were carried out in light of two competing hypotheses that may account for that distribution: the first, due to Everett [21], invokes the adaptive value of ejectives in high-altitude environments because of the reduced articulatory effort of these sounds and/or the advantage in preventing desiccation in the low ambient humidity. The second, alluded to by Nichols [34], rather, operates on considerations having to do with sociolinguistic typology; specifically, sociolinguistic isolation (manifested for example by intra-community use of languages at the highest altitudes and little L2 learning) is thought to lead to the accruing of complex and marked language structures generally, a characterization that applies to both ejectives and uvulars.

Our analyses offer significant improvements in terms of the primary data, i.e. we use the broad cross-linguistic coverage of the PHOIBLE database to follow up on previous work by Everett [21, 30]. We also apply computational phylogenetic approaches to infer the transition rates of ejectives and uvulars on two high-resolution language family phylogenies.

A robustness approach to the analysis of adaptivity of languages to their environment [20], which we adopt, entails, among other things, the test of pertinent hypotheses against different datasets and the use of different statistical methods to assess if, or to what extent, analyses converge on similar results. Rather than a single outcome, this yields a “space of results” that gives an idea of the robustness of the tested hypothesis, which should ideally not be developed ad-hoc, but instead be based on already existing theoretical arguments and experimental evidence. Another hallmark is its reliance on incremental research: by testing already developed hypotheses on a different dataset against which they have not yet been evaluated, and by systematically comparing it with a conceptually different alternative that has not yet been evaluated statistically, our contribution fits squarely within this approach.

On this basis, a Bayesian Mixed Effects Logistic Regression showed that altitude does not have a credible effect on the distribution of uvulars, and only a mild effect on the distribution of ejectives, which is not strongly supported by by-area and by-family treatments. Given this result, we have modelled the phylogenetic evolution of both classes of sounds in two large families of Eurasia (Indo-European and Sino-Tibetan) whose members are spoken in highly heterogeneous environments, and have couched the interpretation of our results in a selective qualitative survey of the pertinent literature. This survey suggests a strong role of language contact in the diachronic development of ejectives in Indo-European and of uvulars in both Indo-European and Sino-Tibetan. It remains to be seen how representative these two case studies are, and identification of further families and application of more rigorous phylogenetic methods are a desideratum for further research to assess the robustness of this result. The situation that emerges from these two case studies, at any rate, is at odds with the scenario that would be predicted by the alternative explanation for the distribution, which instead of arguing for adaptiveness of linguistic structure to environmental givens, would consider altitude as a proxy to sociolinguistic isolation. Both ejectives and uvulars seem to be prone to spread across language boundaries in language contact situations. Similar behavior is noted for clicks and labiovelars [107]: “the evolution or adoption of sounds of these two classes in the sound system of a language is strongly influenced by hearing these sounds in other languages spoken in the same area”, an idea now also captured theoretically [83]. Something similar seems to apply in the case of the two classes of segments which we investigate here. Together with the low support for an effect of altitude on the distribution of uvulars in our Bayesian modelling, our results are inconsistent with the sociolinguistic isolation account, at least in the rather simplistic manner in which it is presently operationalized. But also ejectives have been shown to figure prominently as the targets of replication in contact situations—analogously to the case of Ossetic and, more controversially, Eastern Armenian, they are likely to have been transferred from Aymaran to Quechuan languages in southern Peru and Bolivia in a situation of intense language contact. As Eitan Grossman points out in personal communication, these sounds might be highly perceptually salient, which might give them an advantage in contact situations that offsets their articulatory cost. Together with their particular acoustic effects, this might also make them natural choices as vehicles of iconic motivation, as seems to be the case in the Quechuan-Amyaran contact situation [108, 109]. This is not in principle incompatible with the idea that ejectives are adaptive in high-altitude environments as they may spread in language contact precisely because of their adaptive value. However, as we have noted, before accepting this idea, it would be necessary to specify the relationship between language contact and adaptiveness in a theoretical framework, as this relationship often remains blurry and ambiguous in extant work. Moreover, regarding the Quechuan-Aymaran case, there is strong evidence that the factor that motivated the adoption of ejectives in Quechuan and that facilitated their spread through the native Quechuan lexicon were iconic values associated with them locally [108, 109]. Any argument that would seek to posit adaptiveness as a still more basic principle behind the diachronic developments would be faced with the challenge to factor this fact in.

A final observation that remains to be reiterated is the striking co-occurrence of both classes of sounds, which in fact, was one of the motivating factors for us to consider sociolinguistic isolation as a possible alternative explanation that would account for the distribution of ejectives, but also other types of rare and articulatorily costly segments in the first place. In the case of Ossetic, we have seen that this language has not simply developed ejectives under Nakh-Daghestanian influence, but rather the same characteristic combination of ejectives and uvulars that is found in Nakh-Daghestanian (and Caucasian languages more generally). These cases are representative of a more common cross-linguistic pattern. PHOIBLE contains data from 248 different language families (including isolates as singleton families). Of these, only 68 (circa 28%) feature uvular consonants, and only 65 (circa 26%) ejective consonants, highlighting once more the relative rarity of both classes of speech sounds. There is no logical necessity that both classes of sounds should co-occur in the same languages and language families. However, the distribution of the sounds is strikingly correlated (see also [110]), with a Jaccard Similarity Coefficient of approximately.26 (computed using the *jaccard.test* function as implemented in the R package *jaccard* [111]. Given the overall frequency of language families with the two classes of sounds, this is extremely unlikely to be the result of chance (p < .0000000001).

This suggests that there may actually be common underlying conditioning factors that govern the distributional typology of both classes of sounds and on which it is predicated. What these factors are, and in what terms (typological, sociolinguistic, diachronic, or a combination of these and/or other phenomena) they can be described, is an open and interesting question. As we have mentioned before, it is possible that as yet unknown system-internal factors that shape phonological systems as they expand and are saturated are at play, and we encourage research on these factors. On the other hand, there is an intricate entanglement of socioeconomic organization, the environment and its topography, and speaker behavior on language history and diachronic development. It might well be the interaction of several factors which contribute to a striking clustering of ejectives and uvulars often in the same languages and language families. Whether altitude, either directly though adaptiveness, or indirectly as conducive to sociolinguistic isolation has a (limited) role to play, remains an area for further research to determine.

## Supporting information

S1 Data (TXT)
